# Acute Gastro-Entoritis from an Overdose of Sodium Chloride

**Published:** 1882-07

**Authors:** Geo. M. Parkinson


### V.—ACUTE GASTRO-ENTORITIS FROM AN OVERDOSE OF SODIUM CHLORIDE.

REPORTED BY GEO. M. PARKINSON, D.V.S.

June 4th.—Was called to Middlefield, Ct., to see a cow, aet 7 or 8. Found animal dead.

*Previous History.*—The cow was taken sick in the night, and died in the morning. The evening previous she ate over a quart of rock salt which had been used to make shad brine. The salt, however, had been thoroughly washed. She was in fair flesh.

*Symptoms.*—Bloated rapidly, and gave every evidence of suffering considerable pain; would lie down, then get up, but soon got down and was unable to get up.

I saw her about an hour after she died. She was then lying on her side, and the abdomen was enormously distended with gas.

*Diagnosis*—Gastro-entoritis due to an over indulgence in sodium chloride.

All that was done in the way of treatment was by the owner, who administered some oil.

*Necropsy.*—Two hours after death. There was not the strong sulphuretted hydrogen odor in this case as is common in most cases. *Bladder* distended and contained four or five quarts of urine; but the organ itself appeared to be perfectly normal. *Uterus*, empty. *Rectum*, protruding and congested. Intestines distended by gas, abomasus and small intestines gave evidence of marked inflammation, and at points were black as if gangrene was about to occur. Mesenteric glands slightly enlarged. Small amount of fluid in the peritoneal cavity.

All the stomachs contained a small quantity of ingesta.

MIDDLETOWN, CONN., May, 1882.

